# Impact of residual disease biomarkers on the prognosis of HER2‐positive breast cancer following neoadjuvant therapy

**DOI:** 10.1002/cam4.5839

**Published:** 2023-03-23

**Authors:** Youzhao Ma, Mingda Zhu, Minhao Lv, Peng Yuan, Xiuchun Chen, Zhenzhen Liu

**Affiliations:** ^1^ Department of Breast Disease, Henan Breast Cancer Center The Affiliated Cancer Hospital of Zhengzhou University & Henan Cancer Hospital No.127, Dongming Road Zhengzhou 450008 China

**Keywords:** breast cancer, disease‐free survival, HER2 loss, neoadjuvant therapy, residual disease

## Abstract

**Background:**

The changes in prognostic factors and clinicopathological characteristics of residual disease following neoadjuvant therapy (NAT) for breast cancer are important references for postoperative adjuvant therapy. The analyses of the relationship between the clinicopathological characteristics of residual diseases and the prognosis were not comprehensive in most previous studies. This study aimed to determine how prognostic factors changed following NAT and the impact of clinicopathological characteristics of residual disease on the prognosis of human epidermal growth factor receptor 2 (HER2)‐positive breast cancer.

**Methods:**

The study comprised 350 consecutive patients with HER2‐positive breast cancer who had residual disease after surgery following NAT. The independent risk factors affecting the prognosis of HER2‐positive breast cancer were analyzed using univariate and multivariate Cox regression analyses. Chi‐square test and binary logistic regression were used to analyze the influencing factors of HER2 loss following NAT.

**Results:**

The expression of prognostic factors changed significantly following NAT. A total of 44 patients (12.6%) had HER2 loss. HER2 status (immunohistochemistry (IHC) 3+ vs. IHC 2+/FISH+, *p* < 0.001) at baseline was associated with HER2 loss. In this investigation, the HER2 loss did not affect the prognosis. In univariate analysis, the decrease in Ki‐67 (*p* < 0.001) after NAT was associated with improved prognosis, but this influence was lost in the Cox proportional hazards model. The ypN stage (*p* < 0.001), postoperative ER status (*p* = 0.020), Miller–Payne grade (*p* = 0.007), and targeted therapy (*p* = 0.003) were all independent prognostic factors.

**Conclusions:**

ER, PR, HER2, and Ki‐67 changed significantly after NAT, but no impact of these changes on DFS was observed in this study. Postoperative N stage, postoperative ER status, MP grade, and targeted therapy were independent prognostic factors in patients with HER2‐positive breast cancer after NAT.

## Graphical abstract

The study comprised 350 consecutive patients with HER2‐positive breast cancer who had residual disease after surgery following neoadjuvant therapy. A total of 44 patients (12.6%) had HER2 loss. HER2 status (IHC 3+ vs IHC 2+) at baseline was associated with HER2 loss. The HER2 loss did not affect the prognosis. ER, PR, and Ki‐67 changed significantly after neoadjuvant therapy, but no impact of these changes on DFS was observed in this study. Postoperative N stage, postoperative ER status, MP grade, and targeted therapy were independent prognostic factors in patients with HER2‐positive breast cancer after neoadjuvant therapy. DFS, disease‐free survival; ER, strogen receptor; HER2, human epidermal growth factor receptor 2; IHC, immunohistochemistry; MP grade, Miller‐Payne grade; NAT, neoadjuvant therapy; pCR, pathological complete response; PR, rogesterone receptor.

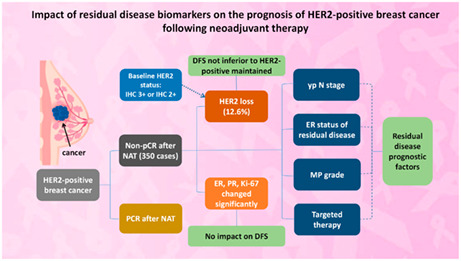

## 1 INTRODUCTION

Human epidermal growth factor receptor 2 (HER2)‐positive breast cancer is one of the most aggressive types of breast cancer. Although not all patients can be cured, anti‐HER2 targeted therapy combined with chemotherapy significantly increases survival in HER2‐positive early breast cancer.^1^, ^2^, ^3^, ^4^ The response of HER2‐positive breast cancer to neoadjuvant therapy (NAT) can serve as a reliable indicator of recurrence risk. Patients who experienced a pathological complete response (pCR) with NAT for HER2‐positive breast cancer have a better prognosis than those who did not.^5^ The selection of postoperative therapy options is also significantly influenced by the effect of NAT. Trastuzumab emtansine can improve the survival of patients with residual invasive disease after NAT.^6^ However, attempts to completely replace trastuzumab in NAT and conventional adjuvant therapy with trastuzumab emtansine failed.^7^, ^8^, ^9^ Therefore, there is still a need for advancements in the treatment of HER2‐positive breast cancer.

Some studies have reported heterogeneity in HER2 expression, which causes resistance to targeted therapy.^10^ As the proportion of NAT gradually increased, we observed changes in the HER2 status of some patients following NAT. We speculate that either this phenomenon is either related to tumor heterogeneity or one of several factors that cause variations in HER2 expression in tumor cells following chemotherapy and targeted therapy. One study observed that residual lymph node disease following NAT may be a better predictor of the prognosis of breast cancer than baseline lymph node status.^11^ The aforementioned topics have been covered in previous studies, however, the majority of them did not comprehensively investigate how the aforementioned factors affected the prognosis. Therefore, the objectives of this study were to compare alterations in clinicopathological features (including HER2 loss) before and after NAT and determine their impact on prognosis in patients with HER2‐positive breast cancer who did not achieve pCR.

## 2 MATERIALS AND METHODS

### 2.1 Patient selection

In this study, we retrospectively analyzed the outcomes of patients with primary breast cancer treated in Henan Cancer Hospital from January 1, 2016, to December 31, 2020. This research was conducted per the standards established in the Declaration of Helsinki. This study was approved by the Medical Ethics Committee of Henan Cancer Hospital (Research Approval Number: 2022–299).

The inclusion criteria were as follows: (1) HER2‐positive breast cancer. HER2‐positive criteria were: HER2 detected by immunohistochemistry (IHC) is 3+ or IHC 2+ and fluorescence in situ hybridization detected with HER2 gene amplification (FISH+). (2) Stage II‐III breast cancer and all patients received NAT. (3) Patients who did not achieve pCR (non‐pCR, residual invasive disease) following NAT, and the residual invasive disease is sufficient for IHC detection. (4) There are accurate data of estrogen receptor (ER), progesterone receptor (PR), Ki‐67 index, and HER2 before and after NAT. (5) Accurate clinical tumor staging is determined by ultrasound or magnetic resonance imaging (American Joint Committee on Cancer (AJCC) systems 7th edition). (6) Clinical nodal staging is determined by palpation of axillary lymph nodes or needle biopsy of suspicious lymph nodes (AJCC systems 7th edition). (7) Pathological tumor and nodal staging are determined by AJCC systems 7th edition. (8) All patients underwent surgery within 3–6 weeks following the completion of NAT, and postoperative pathological data were complete. (9) All patients had complete postoperative therapy information and follow‐up data.

Exclusion criteria: (1) Male breast cancer. (2) Metastatic breast cancer. (3) Bilateral breast cancer. (4) Inflammatory breast cancer. (5) Combined with other primary tumors. (6) Combined with other diseases that cannot tolerate chemotherapy. (7) Incomplete clinicopathological data before and after NAT. (8) The patients with pCR or without sufficient residual invasive disease for IHC detection following NAT.

### 2.2 Information collection and follow‐up

In this study, the clinical data of patients were collected, including age, menopausal status, baseline clinical T and N staging, postoperative pathological T and N staging, preoperative therapy regimen, the expression of ER, PR, and HER2 before and after NAT, date of operation, Miller−Payne grade (MP grade), postoperative pathology, radiotherapy, targeted therapy, relapse time, relapse site, and disease‐free survival (DFS).

In this study, ER ≥1% was defined as positive, and ER <1% was defined as negative; PR ≥1% was defined as positive, and PR <1% was defined as negative; Ki‐67 ≥ 20% was defined as the high expression and Ki‐67 < 20% was defined as low expression. MP grade was primarily used to compare post‐NAT breast tumor specimens and pre‐NAT biopsy specimens for breast cancer. DFS is defined as the period from surgery to disease relapse, death from any cause, or the last follow‐up.

### 2.3 Statistical analysis

Statistical analysis was mainly performed using SPSS 23.0 and R version 4.0.3 in this study. The mean value of continuous variables was compared by T test. The independent risk factors affecting the prognosis of HER2‐positive breast cancer were analyzed using univariate and multivariate Cox regression analyses. Chi‐square test and binary logistic regression were used to analyze the influencing factors of HER2 loss following NAT. In univariate analysis, variables with *p‐*values <0.1 were included in multivariate analysis. Factors with *p*‐values <0.05 in Cox and logistic regression analysis were considered independent prognostic factors. Kaplan–Meier survival curve was used to reflect the impact of independent risk factors on prognosis.

## 3 RESULTS

### 3.1 Patients and tumor characteristics at baseline

**FIGURE 1:**
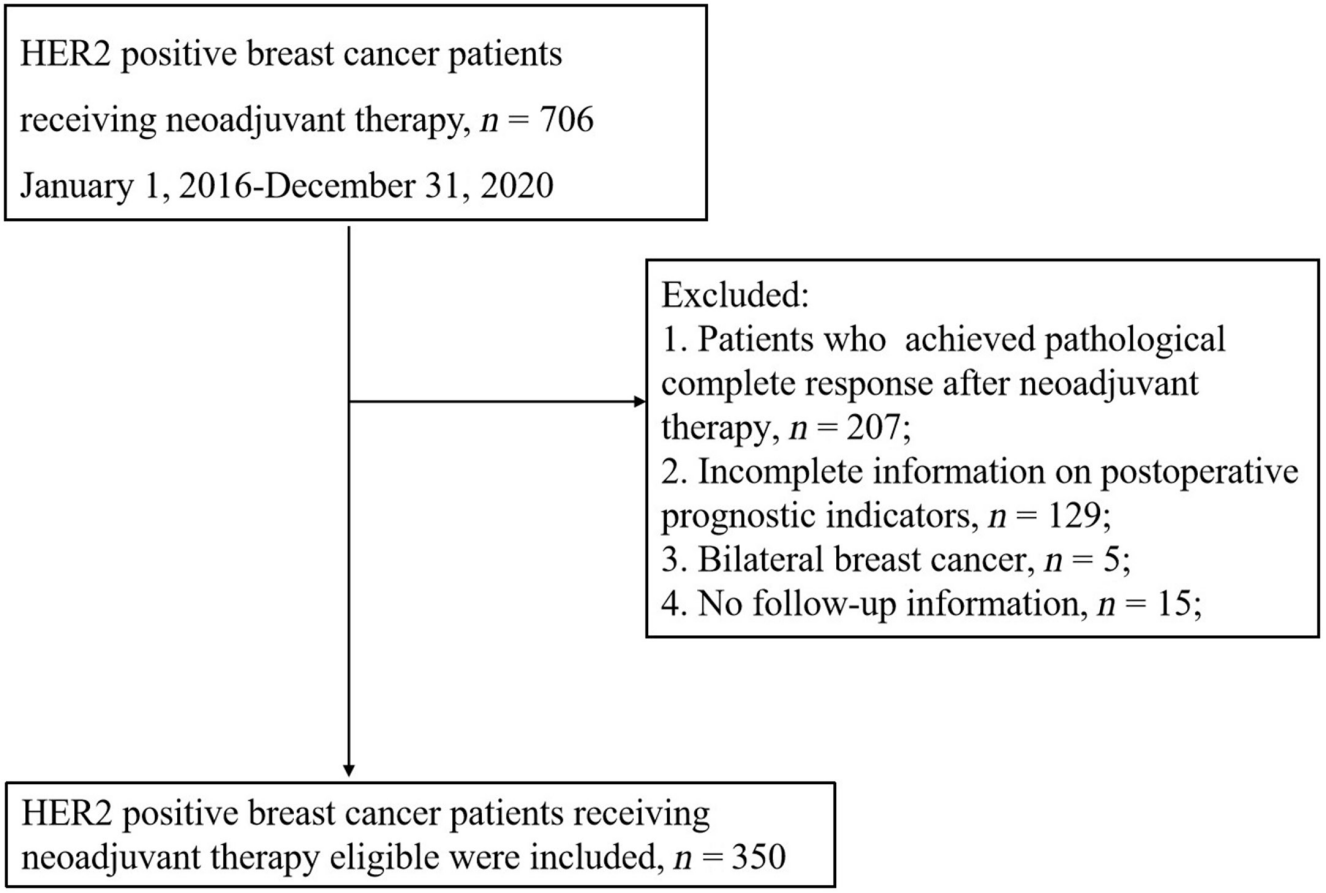
Patient flow diagram.

After excluding patients who did not meet the inclusion criteria, a total of 350 patients with HER2‐positive breast cancer were analyzed in this study (Figure 1). There were 242 patients (69.1%) with HER2 IHC 3+, 108 patients (30.9%) with HER2 IHC 2+ and FISH+, 217 patients (62.0%) with ER+/HER2+, 133 patients (38.0%) with ER ‐/HER2+, 197 patients (56.5%) with PR+/HER2+, and 152 patients (43.5%) with PR −/HER2+. Three hundred and six patients (87.4%) received either trastuzumab or trastuzumab in combination with pertuzumab during NAT (Table 1). Following a median follow‐up of 39 months, DFS events occurred in 76 (21.7%) patients with residual disease after NAT.

**TABLE 1:** Clinicopathological characteristics of the cohort pre‐ and post‐neoadjuvant therapy.

Features	Pre‐NAT	Post‐NAT	*p*‐value
T	*N* (%)	*N* (%)	NA
T1	25	11	
T2	253	220	
T3	56	114	
T4	16	5	
N			NA
N0	50	116	
N1	195	110	
N2	14	61	
N3	91	63	
ER range (mean)	0–100 (44.4 ± 42.5)	0–100 (45.6 ± 42.4)	0.377^a^
PR range (mean)	0–100 (30.2 ± 36.3)	0–100 (19.7 ± 30.1)	<0.001^a^
Ki‐67 range (mean)	2–95 (50.7 ± 20.4)	0–90 (32.9 ± 25.7)	<0.001^a^
HER2			NA
IHC 3+	242 (69.1)	213 (60.8)	
IHC 2+ and FISH+	108 (30.9)	93 (26.6)	
Negative		44 (12.6)	
Targeted therapy			NA
No	44 (12.6)		
Trastuzumab (±pertuzumab)	306 (87.4)		

	Post‐NAT (Negative)	Post‐NAT (Positive)	
pre‐NAT			
ER < 1%	106 (80.9)	25 (19.1)	<0.001^b^
ER ≥ 1%	24 (11.0)	195 (89.0)	
pre‐NAT			
PR < 1%	123 (80.9)	29 (19.1)	<0.001^b^
PR ≥ 1%	54 (27.4)	143 (72.6)	
pre‐NAT			
Ki‐67 <20%	11 (64.7)	6 (35.3)	0.032^b^
Ki‐67 ≥ 20%	126 (38.7)	200 (61.3)	

Abbreviations: ER, estrogen receptor; FISH, fluorescence in situ hybridization; HER2, human epidermal growth factor receptor 2; IHC, immunohistochemistry; NAT, neoadjuvant therapy; PR, progesterone receptor.

^a^ *t*‐test.

^b^ Chi‐square test.

### 3.2 Changes in prognostic factors before and after NAT

**FIGURE 2:**
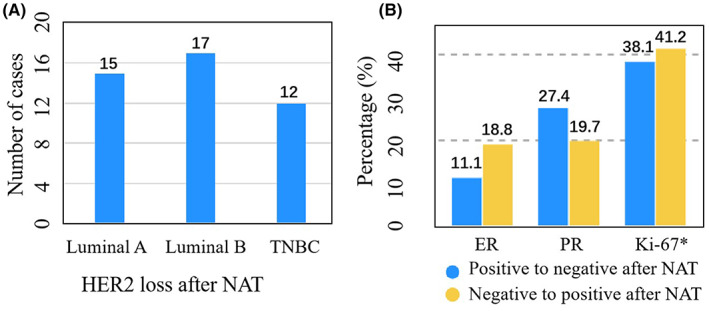
Changes of prognostic factors following neoadjuvant therapy in HER2‐positive breast cancer. (A), Molecular subtype of patients with HER2 loss following neoadjuvant therapy (NAT). (B) Changes of predictors following NAT. TNBC, triple‐negative breast cancer. Ki‐67*, the cut‐off value of Ki‐67 is 20%. Ki‐67 positive to negative means Ki‐67 ≥ 20% before NAT changed to Ki‐67 < 20% after NAT. Ki‐67 negative to positive means Ki‐67 < 20% before NAT changed to Ki‐67 ≥ 20% after NAT.

There were significant statistical differences in the changes in ER, PR, and Ki‐67 index before and after NAT. The expression of Ki‐67 significantly decreased after NAT (*p* < 0.05) (Table 1). A total of 44 patients (12.6%) lost HER2 (from positive to negative) after NAT (Table 2), and 283 patients maintained HER2‐positive. Among the 44 patients experiencing loss of HER2, 15 patients had Luminal A, 17 patients had Luminal B, and 12 patients had triple‐negative breast cancer (TNBC) (Figure 2A). Following NAT, 24 (11.1%) patients lost ER (from positive to negative), and 25 (18.8%) patients gained ER (from negative to positive); 54 (27.4%) patients experienced PR loss and 30 (19.7%) patients experienced PR gain. Prior to NAT, Ki‐67 of 17 patients was <20%, which changed to ≥20% after NAT in 7 patients (41.2%); Ki‐67 of 331 patients was ≥20% before NAT, which changed to <20% after NAT in 126 patients (38.1%) (Table 2) (Figure 2B).

**TABLE 2:** Changes of prognostic factors following neoadjuvant therapy.

Primary tumor (*n*)		Residual disease (*n*)	
HER2 status		HER2 status	
Positive	350	Positive	283 (80.9%)
		Negative	44 (12.6%)
		N/A	23 (6.5%)
ER status		ER status	
≥1%	217	≥1%	193 (88.9%)
		<1%	24 (11.1%)
<1%	133	≥1%	25 (18.8%)
		<1%	108 (81.2%)
PR status		PR status	
≥1%	197	≥1%	143 (72.6%)
		<1%	54 (27.4%)
<1%	152	≥1%	30 (19.7%)
		<1%	122 (80.3%)
Ki‐67 status		Ki‐67 status	
<20%	17	<20%	10 (58.8%)
		≥20%	7 (41.2%)
≥20%	331	<20%	126 (38.1%)
		≥20%	201 (60.7%)
		N/A	4 (1.2%)

Abbreviations: ER, estrogen receptor; HER2, human epidermal growth factor receptor 2; N/A, not available; PR, progesterone receptor.

### 3.3 Clinicopathological features associated with HER2 loss

This study analyzed the clinicopathological features associated with HER2 loss after NAT. In univariate analysis, variables with *p‐*values <0.1 were included in multivariate analysis. Univariate and multivariate analyses revealed that HER2 status (*p* < 0.001) prior to treatment was the influencing factor for HER2 loss following NAT. Patients with pre‐NAT HER2 (IHC 2+) and FISH (+) exhibited a higher rate of HER2 loss after NAT than those with pre‐NAT HER2 (IHC 3+) (25.9% vs 6.6%; odds ratio (OR) = 5.211 [95% CI 2.638–10.294]; *p* < 0.001) (Table 3).

**TABLE 3:** Clinicopathological characteristics associated with a change in HER2 status.

Characteristics	Total	HER2 positive after NAT *N* (%)	HER2 negative after NAT *N* (%)	Univariate Analysis		Multivariate Analysis		
				χ^2^	*p*‐value	OR	95% CI	*p*‐value
Age at diagnosis				2.609	0.106			
≤50	191	162 (84.4)	29 (15.2)					
>50	159	144 (90.6)	15 (9.4)					
Menopausal status				2.761	**0.097**			
Premenopausal	198	168 (84.8)	30 (15.2)			Reference		
Postmenopausal	152	138 (90.8)	14 (9.2)			1.831	0.894–3.749	0.098
cT				0.534	0.911			
T1	25	21 (84.0)	4 (16.0)					
T2	253	223 (88.1)	30 (11.9)					
T3	56	48 (85.7)	8 (14.3)					
T4	16	14 (87.5)	2 (12.5)					
cN				7.343	**0.062**			0.129
N0	50	45 (90.0)	5 (10.0)			Reference		
N1	195	171 (87.7)	24 (12.3)			0.741	0.257–2.137	0.580
N2	14	9 (64.3)	5 (35.7)			0.183	0.004–0.840	0.029
N3	91	81 (89.0)	10 (11.0)			0.794	0.243–2.597	0.703
Pre‐NAT ER				0.024	0.876			
Negative	131	115 (87.8)	16 (12.2)					
Positive	219	191 (87.2)	28 (12.8)					
Pre‐NAT PR				0.143	0.705			
Negative	152	134 (88.2)	18 (11.8)					
Positive	197	171 (86.8)	26 (13.2)					
Pre‐NAT Ki‐67				0.405	0.524			
<20%	17	14 (82.4)	3 (17.6)					
≥20%	331	290 (87.6)	41 (12.4)					
Pre‐NAT HER2				25.345	**<0.001**			
IHC 3+	242	226 (93.4)	16 (6.6)			Reference		
IHC 2+ and FISH+	108	80 (74.1)	28 (25.9)			5.211	2.638–10.294	**<0.001**
Targeted therapy				1.522	0.217			
No	99	90 (90.9)	9 (9.1)					
Yes	251	216 (86.1)	35 (13.9)					

Abbreviations: ER, estrogen receptor; FISH, fluorescence in situ hybridization; HER2, human epidermal growth factor receptor 2; IHC, immunohistochemistry; NAT, neoadjuvant therapy; PR, progesterone receptor.

The bold values are statistically significant values.

### 3.4 Effects of changes in prognostic factors after NAT on DFS

**FIGURE 3:**
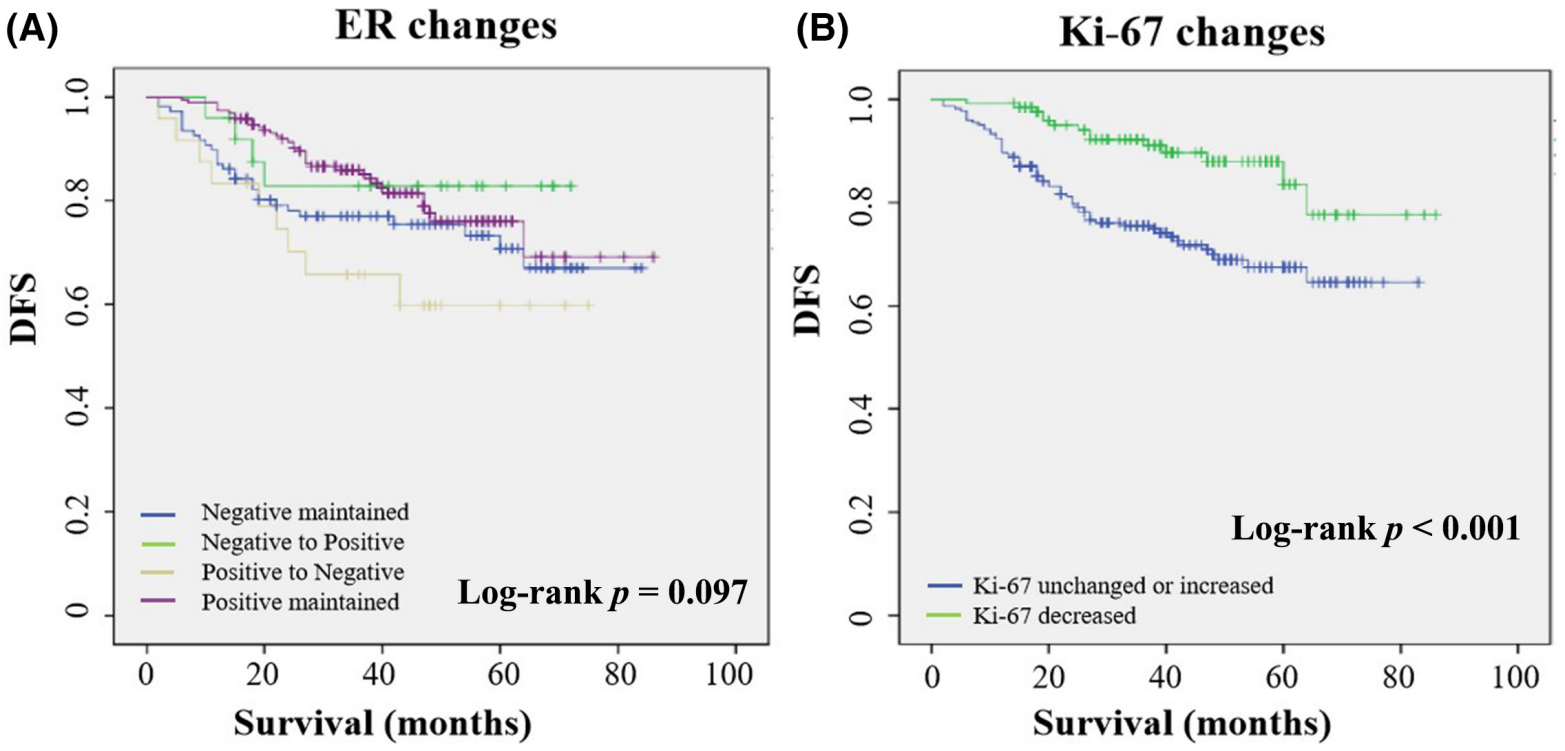
Kaplan–Meier curves for DFS in HER2‐positive breast cancer patients according to ER changes (A) and Ki‐67 changes (B).

After NAT, in univariate analysis, the changes in ER, and PR status were not associated with DFS. There was no significant difference in the DFS between patients who maintained HER2‐positive expression and those who did not. The DFS events in the Ki‐67 decreased group were observed to be lower (11.1%) compared to the Ki‐67 unchanged or increased group (27.9%). The Kaplan–Meier curve of DFS between the two groups showed that Ki‐67 decrease was associated with improved prognosis (log‐rank test *p* < 0.001) (Figure 3; Table 4).

**TABLE 4:** Univariate analysis of the effect of changes in prognostic factors on DFS.

Characteristics	Total	Non‐DFS events *N* (%)	DFS events *N* (%)	Univariate analysis	
				χ2	*p*‐value
ER changes				6.580	0.087
Negative maintained	108 (30.9)	80 (74.1)	28 (25.9)		
Positive maintained	193 (55.1)	158 (81.9)	35 (18.1)		
Negative to Positive	25 (7.1)	21 (84.0)	4 (16.0)		
Positive to Negative	24 (6.9)	15 (62.5)	9 (37.5)		
PR changes				3.675	0.299
Negative maintained	122 (35.0)	89 (73.0)	33 (27.0)		
Positive maintained	143 (41.0)	118 (82.5)	25 (17.5)		
Negative to Positive	30 (8.6)	24 (80.0)	6 (20.0)		
Positive to Negative	54 (15.4)	43 (79.6)	11 (20.4)		
HER2 changes				2.231	0.328
Positive maintained	283 (86.5)	221 (78.1)	62 (21.9)		
Positive to zero	11 (3.4)	7 (63.6)	4 (36.4)		
Positive to low	33 (10.1)	28 (84.4)	5 (15.2)		
Ki‐67 changes				13.178	**<0.001**
No/Increase	219 (63.5)	158 (72.1)	61 (27.9)		
Decrease	126 (36.5)	112 (88.9)	14 (11.1)		

Abbreviations: DFS, disease‐free survival; ER, estrogen receptor; HER2, human epidermal growth factor receptor 2; PR, progesterone receptor.

The bold values are statistically significant values.

### 3.5 The prognostic value of the clinicopathological features of residual disease

**FIGURE 4:**
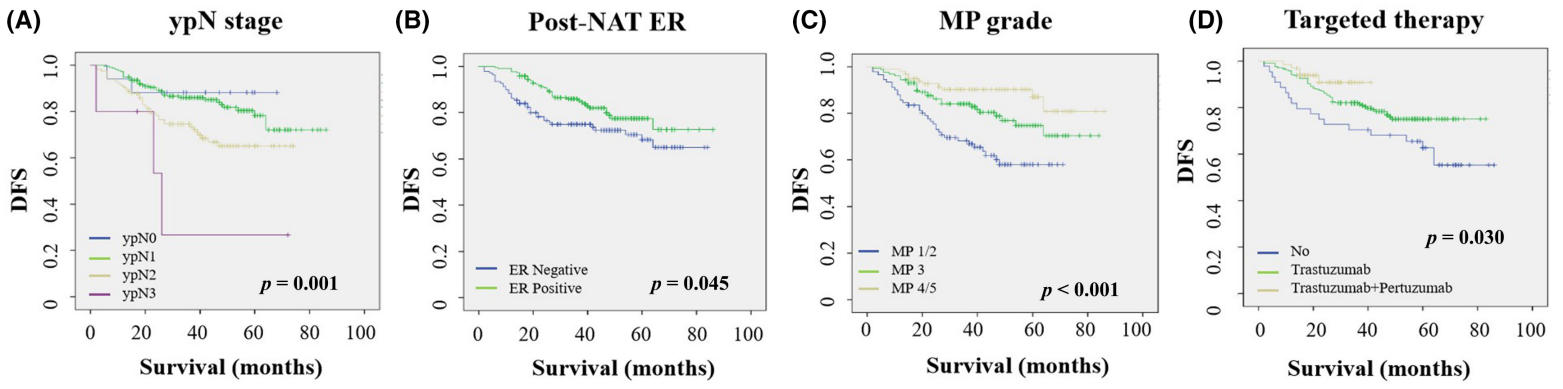
Kaplan–Meier curves for DFS in HER2‐positive breast cancer patients according to ypN stage (A), post‐NAT ER (B), MP grade (C), and targeted therapy (D).

During NAT, 44 (12.6%) patients did not receive targeted therapy, 240 (68.6%) patients received trastuzumab therapy, and 66 (18.8%) patients received trastuzumab with pertuzumab therapy. Univariate analysis and Cox regression analyses revealed that postoperative N stage (*p* < 0.001), postoperative ER status (HR = 0.568 [95% CI 0.350–0.919]; *p* = 0.021), MP grade (*p* = 0.006), and targeted therapy (*p* = 0.003) were independent prognostic factors in patients with HER2‐positive breast cancer following NAT. Neither changes in the Ki‐67 index (*p* = 0.140) nor post‐NAT Ki‐67 (*p* = 0.581) was independent prognostic factor (Table 5). Among patients with HER2‐positive early breast cancer following NAT, those with a lower N stage had a better DFS (log‐rank *p* = 0.001), and those with a higher MP grade had a better DFS (log‐rank *p* < 0.001). ER‐positive patients had a better DFS than ER‐negative patients (log‐rank *p* = 0.045), and patients with dual‐target therapy had a better DFS. Patients without targeted drug therapy exhibited the worst prognosis (log‐rank *p* = 0.030) (Figure 4).

**TABLE 5:** Cox proportional hazards model for postoperative clinicopathological characteristics associated with DFS.

Characteristics	Total	Non‐DFS events *N* (%)	DFS events *N* (%)	Univariate analysis		Multivariate analysis		
				χ2	*p*‐value	HR	95% CI	*p*‐value
Age at diagnosis				0.158	0.691			
≤50	191	148 (77.5)	43 (22.5)					
>50	159	126 (79.2)	33 (20.8)					
Menopausal status				0.000	0.999			
Premenopausal	198	155 (78.3)	43 (21.7)					
Postmenopausal	152	119 (78.3)	33 (21.7)					
ypT stage				11.312	**0.003**			0.203
T0	17	15 (88.2)	2 (11.8)			Reference		
T1	214	178 (83.2)	36 (16.8)			0.898	0.192–5.011	0.892
T2–T3	119	81 (68.1)	38 (31.9)			1.411	0.297–6.704	0.665
ypN stage				30.932	**<0.001**			**<0.001**
N0	116	104 (89.7)	12 (10.3)			Reference		
N1	110	88 (80.0)	22 (20.0)			2.444	1.190–4.985	0.015
N2	61	48 (78.7)	13 (21.3)			2.071	0.918–4.674	0.079
N3	63	34 (54.0)	29 (46.0)			5.406	2.695–10.845	<0.001
Post‐NAT ER				4.348	**0.037**			
Negative	130	94 (72.3)	36 (27.7)			Reference		
Positive	220	180 (81.8)	40 (18.2)			0.568	0.350–0.919	**0.021**
Post‐NAT PR				2.710	0.100			
Negative	178	133 (74.7)	45 (25.3)					
Positive	172	141 (82.0)	31 (18.0)					
Post‐NAT HER2				2.231	0.328			
HER2‐0	11	7 (63.6)	4 (36.4)					
HER2‐low	33	28 (84.8)	5 (15.2)					
HER2‐Positive	306	239 (78.1)	67 (21.0)					
Post‐NAT Ki67				11.628	**0.001**			
<20%	137	120 (87.6)	17 (12.4)			Reference		
≥20%	208	150 (72.1)	58 (27.9)			1.400	0.423–4.636	0.581
Radiotherapy				0.789	0.374			
No	53	39 (73.6)	14 (26.4)					
Yes	296	234 (79.1)	62 (20.9)					
MP grade				20.025	**<0.001**			**0.006**
1–2	91	57 (62.6)	34 (37.4)			Reference		
3	161	130 (80.7)	31 (19.3)			0.546	0.319–0.934	0.027
4–5	98	87 (88.8)	11 (11.2)			0.298	0.136–0.654	0.003
Targeted therapy				17.317	**<0.001**			**0.003**
No	44	26 (59.1)	18 (40.9)			Reference		
Trastuzumab	240	187 (77.9)	53 (22.1)			0.405	0.224–0.732	0.003
Trastuzumab + Pertuzumab	66	61 (92.4)	5 (7.6)			0.217	0.071–0.668	0.008
Ki‐67 changes				13.178	**<0.001**			
No/Increase	219	158 (72.1)	61 (27.9)			Reference		
Decrease	126	112 (88.9)	14 (11.1)			0.385	0.108–1.366	0.140

Abbreviations: DFS, disease‐free survival; ER, estrogen receptor; HER2, human epidermal growth factor receptor 2; MP grade, Miller‐Payne grade; NAT, neoadjuvant therapy; PR, progesterone receptor.

The bold values are statistically significant values.

## 4 DISCUSSION

A huge risk of relapse exists in HER2‐positive breast cancer, particularly in the non‐pCR group following NAT. This study demonstrated a DFS event rate of 21.7% in patients without pCR with a median follow‐up of 39 months, which is similar to the invasive disease‐free survival event rate in the non‐pCR group receiving trastuzumab therapy in the KATHERINE trial with a median follow‐up of 40.9 months.^6^ To determine the parameters impacting prognosis and investigate further therapeutic options to lower the risk of relapse, we collected clinicopathological data from patients with a high risk of relapse before and after NAT and studied their association with prognosis.

Similar to the findings in clinical practice, previous studies have reported that ER, PR, HER2, and Ki‐67 are inconsistently expressed in specimens before and after NAT.^12^ Ki‐67 is an indicator of tumor proliferation index. One study demonstrated that the Ki‐67 values detected by core needle biopsy specimens and postoperative specimens may be inconsistent in patients without NAT.^13^ The changes in Ki‐67 values before and after NAT are evident.^14^ Another study has reported that Ki‐67 decreased after NAT in hormone receptor (HR)‐ and HER2‐positive breast cancers, albeit not in TNBC.^15^ Our study also demonstrated that the average expression level of Ki‐67 in surgical specimens after NAT was significantly reduced compared with the biopsy specimens before NAT. We observed that in univariate analysis the reduction in Ki‐67 values was significantly correlated with the favorable prognosis of patients with HER2‐positive early breast cancer. This was consistent with the conclusions of a previous large‐scale study.^16^ It appears to coincide with the use of Ki‐67 as a predictor of treatment's effectiveness in neoadjuvant endocrine therapy trials, such as in the PALLET trial.^17^ However, the association between Ki‐67 reduction and improved prognosis in this study was not significant in multivariate analysis. In the future, we need a larger sample size to verify this result.

Previous studies have reported that the expression levels of ER, PR, and HER2 in patients without NAT did not significantly change before and after surgery.^13^, ^14^ However, the changes in these predictors owing to NAT were common,^14^, ^18^, ^19^, ^20^ of which those in PR expression levels may be the most prevalent. Consequently, the adjuvant therapy of some patients was changed. Variations in the initial Luminal subtype were the most prevalent; however, they had the least influence on the modification of adjuvant therapy. Changes in initial HR‐ and HER2‐negative patients had a greater impact on the change in adjuvant therapy. Our study yielded similar findings. Our study revealed that the ER gain incidence was higher than ER loss incidence. This is consistent with the findings of Renu Gahlaut et al.^19^ Endocrine therapy was recommended if ER/PR was positive in our study's bioptic or postoperative sample. Consistent with the findings of a previous study,^21^ we observed that the impact of ER changes on the prognosis was not statistically significant. Patients with ER loss have a worse DFS than patients with ER‐positive or ER‐negative maintained and ER gain. These findings suggest that residual disease predictors after NAT may have a greater impact on prognosis. These results support the popular perception that patients receiving endocrine therapy had a better chance of survival.

This study focused on variations in HER2 status. In daily clinical practice, most patients whose HER2 status had changed from positive to negative after NAT still received targeted therapy with trastuzumab (or trastuzumab in combination with pertuzumab), especially those who were unable to use trastuzumab emtansine (owing to drug toxicity or economic reasons). In some studies, patients who experienced HER2 loss after NAT had a worse prognosis than those who maintained HER2‐positive.^22^, ^23^, ^24^ This may be attributed to tumor heterogeneity, and HER2‐positive cancer cells can be eliminated by targeted therapy whereas HER2‐negative cancer cells cannot be eliminated owing to their insensitivity to targeted therapy. However, there was no statistically significant difference between the survival of patients who lost HER2 and those who maintained HER2 at a median follow‐up of 39 months in this study. We considered that this difference may be attributed to the small proportion of patients experiencing HER2 loss and the shorter follow‐up duration of this study. Thus, these results also suggest that we must consider whether it is appropriate to continue trastuzumab therapy in patients with HER2 loss during the adjuvant therapy phase following NAT. After NAT, trastuzumab emtansine is currently the preferred therapy option for patients who were non‐pCR HER2‐positive, albeit the level of evidence‐based medicine for this recommendation in patients receiving dual‐target therapy in the NAT phase was not considerably high owing to the proportion of the enrolled population in the KATHERINE trial. Prospective studies have demonstrated that trastuzumab deruxtecan can improve the prognosis of patients with HER2‐low advanced breast cancer.^25^ Thirty‐three out of 44 patients with HER2 loss exhibit HER2‐low expression in our study. Trastuzumab deruxtecan may improve the prognosis of these patients.

In this study, the proportion of HER2 loss in the neoadjuvant chemotherapy combined with the targeted therapy group was higher than that in the chemotherapy alone group, albeit there was no statistical difference. In the aforementioned studies, the proportion of HER2 loss in the chemotherapy combined with the targeted therapy group versus the chemotherapy alone group varied.^22^, ^23^ These results suggest that several factors may have an impact on alterations in HER2 status both before and after NAT. In this study, the contributing elements leading to HER2 loss were analyzed. It is noteworthy that the initial HER2 expression status (IHC 3+ versus IHC 2+/FISH+) had a greater impact on HER2 changes, suggesting that patients with poor HER2 expression (IHC 2+/FISH+) among HER2‐positive patients may be more susceptible to HER2 loss. We speculated that the heterogeneity of HER2 expression may be involved. The heterogeneity of HER2 expression may impact the efficacy of targeted therapy, further affecting prognosis. Differences in detection methods may also affect the accuracy of HER2 status interpretation. Studies have revealed that the inconsistent HER2 expression level detected by IHC and in situ hybridization is 4%–9%.^26^ This is most likely one of the reasons for the disparities in HER2 loss proportions across studies.

This study demonstrated that the clinicopathological characteristics of postoperative residual disease after NAT had a greater impact on prognosis. Low postoperative regional lymph node stage, positive ER, high MP grade in breast lesions, and targeted therapy are effective prognostic markers. One study has also revealed that the lymph node status and the Ki‐67 values of residual disease affect prognosis after NAT.^27^ In our study, the decrease in Ki‐67 values after NAT and the Ki‐67 values of residual disease did not affect the prognosis. This may be associated with the different study designs and cut‐off values of Ki‐67. The impact of lymph node staging on prognosis indicates the importance of accurate axillary staging after NAT. We must be cautious when deciding to forgo axillary dissection following NAT for patients with HER2‐positive early breast cancer. ER status is a prognostic indicator of postoperative residual disease that is still valuable to retest. In this study, the change in the HER2 expression level of postoperative specimens did not affect the prognosis, most likely owing to the small sample size of postoperative specimens with HER2 loss. Dual‐target therapy had a greater effect than single‐target therapy, and single‐target therapy had a greater effect than no targeted therapy. These results suggest that continuation of trastuzumab + pertuzumab (HP) dual‐target therapy can improve survival in non‐pCR patients even when trastuzumab emtansine is difficult to obtain or has intolerable side effects. Although the prognostic value of T staging has not been established, MP grading, which represents the shrinkage of the primary tumor to some extent, has a significant impact on prognosis. This suggests that the impact of the primary tumor on the prognosis was not determined by the preoperative or postoperative staging but by the degree of tumor degeneration, including the level of decrease in cancer cell density. The clinicopathological characteristics of residual disease following NAT and surgery must therefore be prioritized.

The advantage of this study is the large sample size and the collection of a dataset of clinicopathological characteristics of residual invasive disease in patients with HER2‐positive early breast cancer after NAT. This study analyzed the relationship between clinicopathological characteristics of residual disease and survival after NAT. The main limitation of this study was that it was a single‐center retrospective study. The data of this retrospective study were from a single‐center database and needs to be verified by multi‐center data. It also has the same selective bias as other retrospective studies. Due to drug availability and economic reasons, most of the patients in this study did not receive dual‐target therapy in the neoadjuvant setting, and no patients received trastuzumab emtansine in adjuvant setting. Therefore, the conclusions of this study may not be applicable to the population who received dual‐target therapy in neoadjuvant setting or trastuzumab emtansine in adjuvant setting. This is one of the limitations of this study. Pathological data were obtained from the interpretation of pathologists in daily treatment, and there may be a deviation. The median follow‐up time was only 39 months, which requires a longer follow‐up duration to verify our conclusions. The lack of overall survival data was another limitation of this study.

## 5 CONCLUSION

In conclusion, ER, PR, HER2, and Ki‐67 changed significantly after NAT, but no impact of these changes on DFS was observed in this study. The intensity of HER2 expression may be the most important influencing factor of HER2 loss. The clinicopathological characteristics of residual disease after NAT have a significant impact on prognosis. Postoperative N stage, postoperative ER status, MP grade, and targeted therapy were independent prognostic factors in patients with HER2‐positive breast cancer after NAT.

## AUTHOR CONTRIBUTIONS

**Youzhao Ma:** Conceptualization (lead); writing – original draft (lead). **Mingda Zhu:** Formal analysis (lead); visualization (lead). **Minhao Lv:** Data curation (equal). **Peng Yuan:** Data curation (equal). **Xiuchun Chen:** Supervision (equal); writing – review and editing (equal). **zhenzhen liu:** Conceptualization (equal); writing – review and editing (equal).

## FUNDING INFORMATION

This work was supported by a grant from Henan province medical science and technology research project (LHGJ20220204).

## CONFLICT OF INTEREST STATEMENT

No potential competing interests are disclosed.

## ETHICS APPROVAL STATEMENT

This research was conducted in accordance with the standards set out in the Declaration of Helsinki. This study was approved by the Medical Ethics Committee of Henan Cancer Hospital (Research Approval Number: 2022–299). The Medical Ethics Committee of Henan Cancer Hospital did not require patients to agree to review their medical records (On the premise of not disclosing the privacy of patients, doctors can use it for clinical research).

## Data Availability

The datasets used and/or analyzed during the current study are available from the corresponding author upon reasonable request.
